# Safety Assessment of Liver-Targeted Hydrodynamic Gene Delivery in Dogs

**DOI:** 10.1371/journal.pone.0107203

**Published:** 2014-09-24

**Authors:** Kenya Kamimura, Tsutomu Kanefuji, Takeshi Yokoo, Hiroyuki Abe, Takeshi Suda, Yuji Kobayashi, Guisheng Zhang, Yutaka Aoyagi, Dexi Liu

**Affiliations:** 1 Division of Gastroenterology and Hepatology, Graduate School of Medical and Dental Sciences, Niigata University, Niigata, Japan; 2 Department of Pharmaceutical and Biomedical Sciences, College of Pharmacy, University of Georgia, Athens, Georgia, United States of America; Saitama Medical University, Japan

## Abstract

Evidence in support of safety of a gene delivery procedure is essential toward gene therapy. Previous studies using the hydrodynamics-based procedure primarily focus on gene delivery efficiency or gene function analysis in mice. The current study focuses on an assessment of the safety of computer-controlled and liver-targeted hydrodynamic gene delivery in dogs as the first step toward hydrodynamic gene therapy in clinic. We demonstrate that the impacts of the hydrodynamic procedure were limited in the injected region and the influences were transient. Histological examination and the hepatic microcirculation measurement using reflectance spectrophotometry reveal that the liver-specific impact of the procedure involves a transient expansion of the liver sinusoids. No systemic damage or toxicity was observed. Physiological parameters, including electrocardiogram, heart rate, blood pressure, oxygen saturation, and body temperature, remained in normal ranges during and after hydrodynamic injection. Body weight was also examined to assess the long-term effects of the procedure in animals who underwent 3 hydrodynamic injections in 6 weeks with 2-week time interval in between. Serum biochemistry analysis showed a transient increase in liver enzymes and a few cytokines upon injection. These results demonstrate that image-guided, liver-specific hydrodynamic gene delivery is safe.

## Introduction

Gene therapy is one therapeutic option, with on-going clinical trials, for treating various diseases [1]. Assessment of the safety and effectiveness of a gene delivery method is an essential step toward clinical applications. Among gene delivery procedures established thus far, the hydrodynamic gene delivery method was originally developed as a simple and efficient method to transfer genes to the mouse liver by rapid tail vein injection of a large volume of DNA solution equal to 9% of body weight [2], [3]. Hydrodynamic gene delivery has been studied extensively for the functional analysis of genetic elements, siRNA-based target validation, establishment of animal models for viral infection and protein drug discovery (see recent reviews [4]–[7]). Since this procedure is highly effective and does not require a vector, efforts have been made to apply this procedure for gene therapy in large animals. A few groups, including ours, have reported successful hydrodynamic gene delivery in rabbits and pigs [8], [9]. We have developed and demonstrated the efficacy of a computer-controlled, image-guided, and liver-specific hydrodynamic procedure in pigs [6], [8], [10]. More recently, we have demonstrated that the procedure can be safely applied to large pigs with a body weight of 40–65 kg, close to that of a man. Parameters critical to successful gene delivery include the location of the balloon catheter in the hepatic vein, intravascular pressure upon injection, injection volume, and sequential injections to multiple lobes of the injected liver [11]. To date, the safety of the procedure has been assessed only by biochemical analysis because the primary objective of the previous studies was to assess gene delivery efficiency. A comprehensive analysis of the procedure regarding its short-term and long-term safety concerns is necessary prior to in clinic application of this simple and convenient procedure. Therefore, the goal of the current study is to assess the safety of the organ-specific, regional hydrodynamic gene delivery to demonstrate the clinical applicability of the hydrodynamic gene delivery. The assessments on the short- and long-term impacts of the procedure using various physiological, histological, and biochemical analysis were conducted in dogs, an animal model that exhibits more similarity in liver structure to that of humans than that of rabbits or pigs. We demonstrated that the impacts of image-guided liver-targeted hydrodynamic gene delivery are localized in the injected liver section with significant expansion of sinusoids. No respiratory, cardiovascular, or long-term body weight changes were observed throughout the study. For the first time, we also explored the levels of the cytokines following the hydrodynamic and systemic injections to large animals. Interestingly, the results showed no increase in the systemic inflammatory cytokines of IFN-γ, IL-8, IL-18, and IL-4, but did show an increase in TNF-α, IL-10, MCP-1, canine KC, and IL-6, which were related to vascular stretching. Our results strongly support the safety of the image-guided, liver-specific hydrodynamic gene delivery and its clinical applicability.

## Materials And Methods

### Materials

The pCMV-Luc plasmid, containing firefly luciferase cDNA driven by a CMV promoter and the pCAG-hAAT plasmid, containing human alpha-1 antitrypsin gene driven by a chicken beta-actin promoter with CMV enhancer, were purified by the double CsCl–ethidium bromide gradient centrifugation method and was kept in a Tris–EDTA buffer. The pBS-HCRHP-FIXIA plasmid, containing the human factor IX gene, driven by the hAAT promoter, was kindly provided by Dr. Carol Miao's lab [12]-[14]. The purity of the plasmid preparation was checked by absorbency at 260 and 280 nm and 1% agarose gel electrophoresis. The introducer and the 9 Fr sheath for image-guided catheter insertion were from COOK (Bloomington, IN, USA) and the guide wire (ZIP wire) was from Boston Scientific (Natick, MA, USA). Injection balloon catheters (9 Fr) were purchased from the Tokai Medical Co., Ltd. (Kasugai, Aichi, Japan). The pressure transducer was from St. Jude Medical Japan Co., Ltd. (Minato-ku, Tokyo, Japan). The contrast medium (PROSCOPE) was from Mitsubishi Tanabe Pharma (Cho-ku, Osaka, Japan). WavelineVet Vital Signs Monitor for monitoring physiological parameters on animals was from DRE Veterinary (Louisville, KY, USA). Hound dogs (female, 15–20 kg) were from either Marshall Bioresources (North Rose, NY, USA) or Kitayama Labs Co., LTD. (Ina, Nagano, Japan). Wistar rats (female, 150–200 g) were purchased from Japan SLC, Inc. (Hamamatsu, Shizuoka, Japan).

### Ethics statement

All animal experiments were conducted in full compliance with regulations and approved by the Institutional Animal Care and Use Committee at the Niigata University, Niigata, Japan (#163-5) and the University of Pittsburgh, Pittsburgh, Pennsylvania, USA (#0808112).

### Animal procedure

Under general anesthesia using isoflurane, hound dogs were placed on the table of fluoroscopy machine YSF-100 from Shimadzu Corp. (Nakagyo-ku, Kyoto, Japan). An 18G peripheral catheter (TERUMO, Sibuya-ku, Tokyo, Japan) was directly inserted into the jugular vein followed by insertion of a 0.035-inch hydrophilic guide wire, a short sheath (9 Fr), and an injection balloon catheter. The balloon was inflated by injecting 1 ml of phase contrast medium, and the obstruction of the blood flow was verified by injecting a small volume of phase contrast medium into the vasculature through the catheter. Rats were anesthesized using isoflurane and 2,2,2–tribromoethanol (concentration, 0.016 g/ml in 0.9% saline; dose, 1.25 ml/100g body weight). Through a small incision in the abdominal wall, an injection catheter (SURFLO 22 gauge, TERUMO, Sibuya-ku, Tokyo, Japan) was inserted *via* the inferior vena cava (IVC), and its tip was placed at the junction of the IVC and the hepatic veins. Saline was hydrodynamically injected into the liver with temporal blood flow occlusions at the supra- and infra-hepatic IVC, as previously reported [15].

### Hydrodynamic injection

Image-guided, liver-targeted hydrodynamic gene delivery was performed in dogs (n = 8) using the computer-controlled injection system as previously reported [8], [11], [16]. In brief, an injection of 2.5× volume of each of the 4 liver lobes, the right lateral, right medial, left medial, and left lateral lobe (approximately 250 ml for each lobe in 20 kg dogs) of either saline, or saline containing plasmid DNA (100 µg/ml) (n = 2 for each injection with saline, pCAG-hAAT, pBS-HCRHP-FIXIA, or pCMV-Luc plasmids) was performed within 12 sec sequentially to all 4 lobes through the hepatic veins in all dogs with 20 min of interval between each injection (in total, ∼1,000 ml was injected within 1 h). The same setting was used for the 2^nd^ and 3^rd^ sets of hydrodynamic injections in the same dogs with a 2-week interval in between. Various physiological parameters including heart rate (HR), systolic blood pressure (SBP), diastolic blood pressure (DBP), oxygen saturation (SpO2), body temperature (BT), and electrocardiogram (ECG) were continuously monitored and recorded before, during, and after each injection. For experimental control, a slow drip infusion of total of 1,000 ml of saline was performed from the cephalic vein within either 1 h (∼0.28 ml/sec) or 2 h (∼0.14 ml/sec) in 2 dogs. The liver-targeted hydrodynamic injections to rats were performed as previously described [15]. Briefly, a midline skin incision was made under general anesthesia. With a small incision in the abdominal wall, an injection catheter (SURFLO 22 gauge; Terumo, Sibuya-ku, Tokyo, Japan) was inserted via the IVC and its tip placed at the junction of the IVC and the hepatic veins. The steady manual injection of 6% BW saline in 10 s (∼1.2 ml/sec) was performed to the liver of a 200 g rat with temporal blood flow occlusions at the supra- and infrahepatic IVC. The incision made in the animals' abdomen was sutured after the procedure.

### Immunohistochemical staining

Dogs were euthanized, and the liver samples were collected for luciferase staining 24 h after completion of all hydrodynamic injections to each of the 4 lobes of the dog with pCMV-Luc plasmid DNA. Tissue samples were fixed in 10% formalin upon collection and embedded in paraffin. Sections (10 µm) were made, and standard immunohistochemistry was performed with a goat anti-luciferase polyclonal antibody (G7451, 1∶50 dilution; Promega, Madison, WI), Vecstatin Elite ABC Goat IgG Kit (PK–6105; Vector Laboratories Inc., Burlingame, CA), and DAB chromogen tablet (Muto Pure Chemicals CO., Ltd, Bunkyo-ku, Tokyo).

### Measurement of the hepatic microcirculation

The light-conducting fiberoptic bundle was gently placed on the surface of the right medial lobe of the rat liver during the liver-targeted hydrodynamic injection to the isolated liver. The index of blood volume in regional hepatic tissue [IHB] and the index of blood oxygenation in regional hepatic tissue [ISO_2_] were monitored using a reflectance spectrophotometry system (TS-200, Sumitomo Electric, Osaka, Japan) before, during, and after hydrodynamic injection at a constant flow rate of 1.2 ml/sec as previously reported [17].

### Assessment of tissue damage

Blood samples were collected from each dog before (time  = 0), and 2, 4, 24, 48, and 96 h after either hydrodynamic injection or slow injection (1,000 ml) in 1 or 2 h through the peripheral vein as controls. The serum biochemical analysis and hemogram were performed by the Cleveland Office of Marshfield Labs and BML Inc. (Shibuya-ku, Tokyo, Japan). The cytokines were analyzed by the GeneticLab. Co. Ltd. (Sapporo, Hokkaido, Japan) using the Luminex 200 System with the MILLIPLEXMAP Canine Cytokine/Chemokine Kit (MILLIPORE, CCYTO-90K) [18], [19]. Tissue samples for hematoxylin and eosin (H&E) staining were collected before, immediately following, and 3 h after the hydrodynamic injection to rats, and before, immediate following, and 24 h after the injection to dogs. Three different liver sections from each of the 3 rats and 2 dogs were stained, images were captured, and a quantitative analysis of the sinusoidal area was performed using ImageJ software (version 1.6.0_20, National Institutes of Health, USA) as previously reported [20].

### Statistical methods

The tissue toxicity assays were statistically evaluated by ANOVA and Bonferroni's multiple comparison test.

## Results

### Effect of the liver targeted hydrodynamic gene delivery and impacts on animals

**Figure 1:**
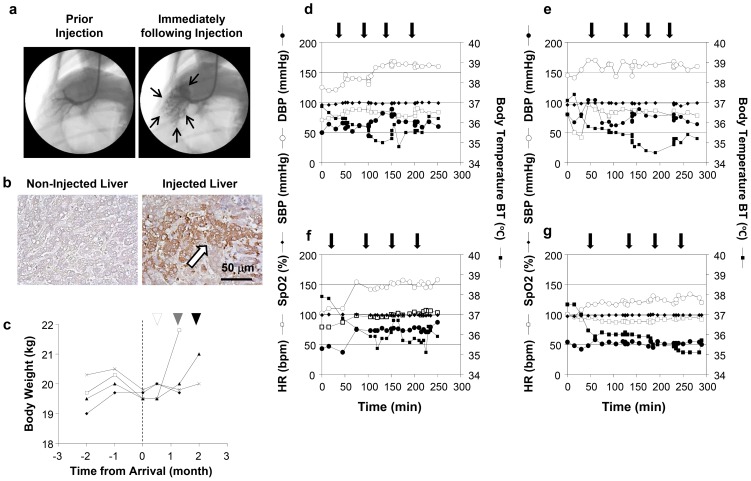
Liver-targeted hydrodynamic gene delivery. (**a**) Location of the balloon catheter in the hepatic vein. A small amount of contrast medium was injected into the vein to confirm the occlusion of the blood flow and distribution of injected solution during a liver lobe-specific hydrodynamic gene delivery. Black arrows represent the distribution of the contrast medium in the injected liver lobe. (**b**) Immunohistochemical staining of the liver. Liver samples from control and plasmid DNA injected liver were stained with anti-luciferase antibodies. Scale bar represents 50 µm. White arrow indicates the positively stained hepatocytes. (**c**) The change of body weight before, after the arrival of the animal to the animal facility. White, gray, and black arrowheads point the hydrodynamic injections in representative 4 dogs. (**d–g**) Physiological impacts of the sequential, liver-targeted hydrodynamic gene delivery in representative 4 dogs. Physiological parameters before, during, and after the sequential liver-targeted hydrodynamic injection of saline (**d**), pCMV-Luc (**e**), pCAG-hAAT (**f**), or pBS-HCRHP-FIXIA (**g**). HR, heart rate (white square); SBP, systolic blood pressure (white circle); DBP, diastolic blood pressure (black circle); SpO_2_, oxygen saturation (black diamond); BT, body temperature (black square). Black arrows indicate each of the 4 lobe injections.

**Figure 2:**
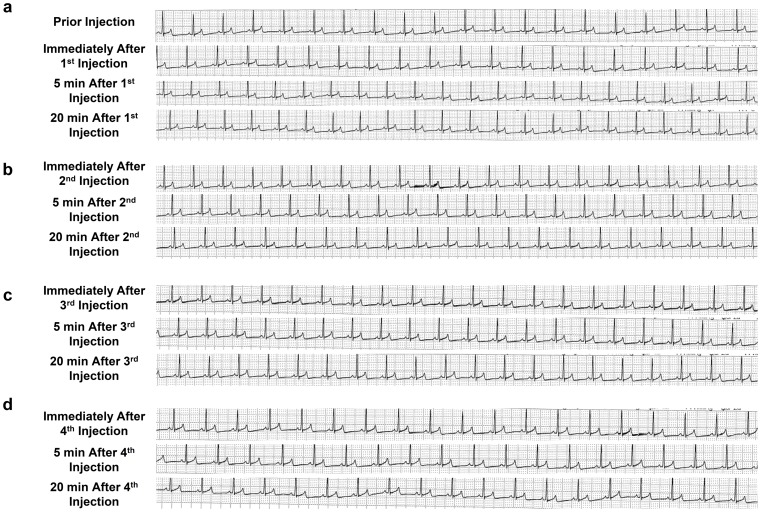
Electrocardiograms of animals prior to, immediately following, and 5 and 20 min after each of 4 lobe-specific injections. The electrocardiogram was monitored before, immediately following, 5 min, and 20 min after each of injection of pCMV-Luc to right lateral lobe (a), right medial lobe (b), left medial lobe (c), and left lateral lobe (d) in dog 1 with 20 min of interval time between 2 injections.

The physiological impacts of the image-guided and liver-targeted hydrodynamic gene delivery procedure were examined in dogs. The distribution of the contrast medium was confirmed before gene delivery to ensure appropriate catheter placement (**Figure 1a**). The intravascular pressure reached 110 mmHg from the baseline venous pressure during the injection (data not shown). Gene delivery efficiency was assessed by immunohistochemical staining of liver samples collected 24 h after hydrodynamic delivery of pCMV-Luc plasmid DNA (**Figure 1b**). Approximately 10–15% of hepatocytes were stained positive with the anti-Luc antibody in all 4 lobes injected. To examine procedure- and injection material-related toxicity, either saline or saline with plasmids pCAG-hAAT, pBS-HCRHP-FIXIA or pCMV-Luc, was hydrodynamically injected into all 4 lobes individually of dogs (2 dogs for each). The animals were bright, alert, responsive, defecating, urinating, drinking, and eating very well in our animal facility soon after the procedure and in the following days. Other than a slight decrease in the body weight of the dogs after the long-distance transfer from the field to our animal facility, no significant decrease in body weight was seen throughout the 3 rounds of injections with 2-week interval in between (**Figure 1c**). No chronic vascular damages were seen in hepatic veins with fluoroscopy and distribution of the contrast medium. The 2^nd^ and 3^rd^ injection showed no significant difference from that of the 1^st^ injection. No edema or ascites were observed in any animals upon necropsy. Physical examinations, including heart rate (HR), systolic blood pressure (SBP), diastolic blood pressure (DBP), oxygen saturation (SpO_2_), body temperature (BT), (**Figure 1d–g**), and electrocardiogram (ECG) (**Figure 2**), were monitored before, during, and after hydrodynamic injection. Body temperature dropped 6.7% from the baseline to the lowest level at 34.5°C after injections and returned to baseline level 3 h after the last injection. This may be attributed to the relatively low temperature solution used (∼30°C and kept in the reservoir placed in the room temperature). A slight increase in SBP after the injection was also recorded. No changes in HR, DBP, and SpO_2_ were observed in any of the 4 dogs injected (**Figures 1d–g**). The ECG showed no changes in the dynamic ST segment and T wave changes while performing sequential hydrodynamic injection to each of the 4 hepatic veins with the optimum parameters (representative data from injections 1–4 in 1^st^ study in dog 1 are shown in **Figure 2**). These results suggest that efficient, liver-specific hydrodynamic gene delivery has no significant impact on physiological parameters, such as systemic circulation, respiration, and cardiac function.

### Impact of the liver-targeted hydrodynamic gene delivery on hepatic tissue

**Figure 3:**
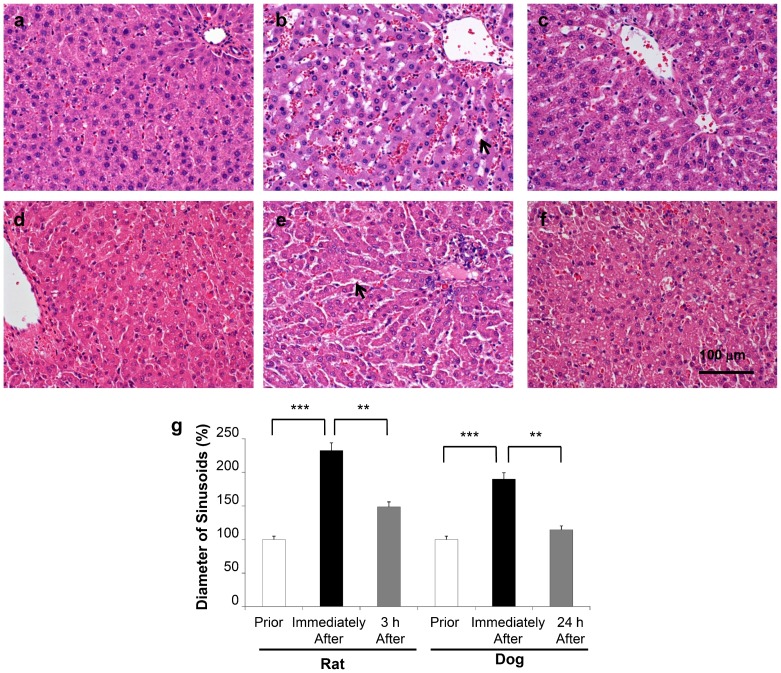
Impact of liver-targeted hydrodynamic gene delivery on hepatic sinusoidal structure. Hematoxylin and eosin staining was performed on the liver sample before (**a**), immediately following (**b**), and 3 h (**c**) after the liver-targeted hydrodynamic gene delivery in rats, and prior to (**d**), immediately following (**e**), and 24 h (**f**) after the liver-targeted hydrodynamic gene delivery in dogs. Scale bar represents 100 µm. (**g**) Quantitative measurement of hepatic sinusoidal areas. Three different liver sections from 3 rats (n = 9) and 2 dogs (n = 6) were stained and images were captured. Quantitative analysis was performed using ImageJ software (version 1.6.0_20, National Institutes of Health, USA). The values represent mean ± SD. ** *p*<0.01, *** *p*<0.001 by ANOVA and Bonferroni's multiple comparison test.

To examine the impact of liver-targeted hydrodynamic gene delivery on liver tissue, a histological analysis was performed on the samples collected before and after injections at the appropriate time points in rats (**Figures 3a–c**) and dogs (**Figures 3d–f**). H&E staining showed significant expansion of the sinusoids, up to 232% in rats (**Figures 3b, 3g**) and 190% in dogs (**Figures 3e, 3g**), from their original area (**Figures 3a, 3d**) immediately following the liver-targeted hydrodynamic injection. The expansion returned to 148% (**Figures 3c, 3g**) and 114% (**Figures 3f, 3g**) in 3 and 24 h, respectively, after the hydrodynamic procedure, in each animal. These results suggest that liver-targeted hydrodynamic gene delivery resulted in the transient expansion of the sinusoidal structure in both rats and dogs.

### Impact of the liver-targeted hydrodynamic gene delivery on hepatic microcirculation

**Figure 4:**
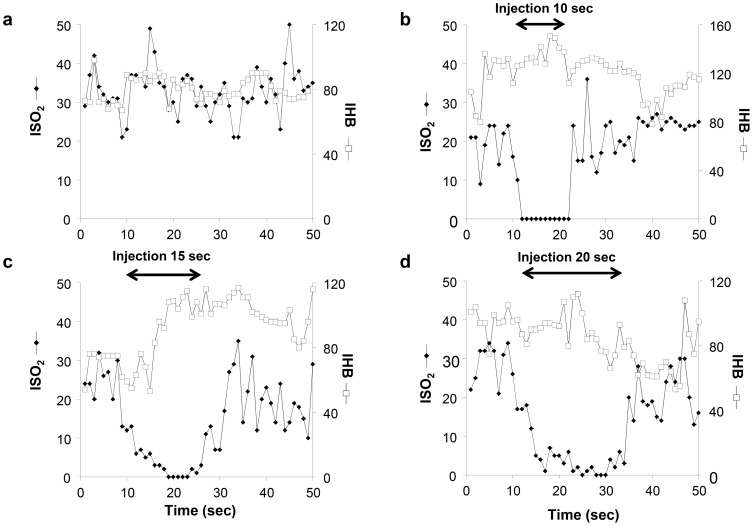
Impact of liver-targeted hydrodynamic gene delivery on hepatic microcirculation. The total blood volume (IHB, white square) and the blood oxygenation (ISO_2_, black diamond) in regional hepatic tissue under normal conditions (**a**), prior to, during, and after the liver-targeted hydrodynamic saline injection in 10 sec (**b**), 15 sec (**c**), and 20 sec (**d**) to rat livers.

To examine the impact of liver-targeted hydrodynamic gene delivery on hepatic microcirculation and to confirm the effect of the injected solution on the sinusoidal structures, microcirculation in the liver was monitored in rats by reflectance spectrophotometry during the injection (**Figure 4**). The Hb content of the liver (IHB), reflecting the hepatic blood volume *in situ*, and the oxygen saturation of the Hb in the blood of regional hepatic sinusoids (ISO_2_) were monitored in non-injected control rats (**Figure 4a**) and in rats receiving liver-targeted hydrodynamic injection with different injection time and volume (**Figures 4b–d**). The light-conducting fiberoptic bundle was placed on the hepatic surface of the lobes during the injections. The IHB showed no significant changes during the injections in 10, 15, and 20 sec with a flow rate of 1 ml/sec (**Figures 4b–d**), the same setting used in the previous experiment (**Figures 3a-c**). In contrast, the ISO_2_ showed a significant decrease in the oxygenation from the normal level at ∼20 units to zero during the period of hydrodynamic injection, and recovered within 10 sec after injection (**Figures 4b–d**), suggesting that the injected solution has been pushed into the sinusoid, diluted the blood and expanded the area, so that the DNA can be exposed to liver. These results support the conclusion drawn from the histological analyses (**Figures 3a–c**). The non-injected rats showed no change in IHB or ISO_2_ (**Figure 4a**).

### Impact of liver-targeted hydrodynamic gene delivery on serum biochemistry

**Figure 5:**
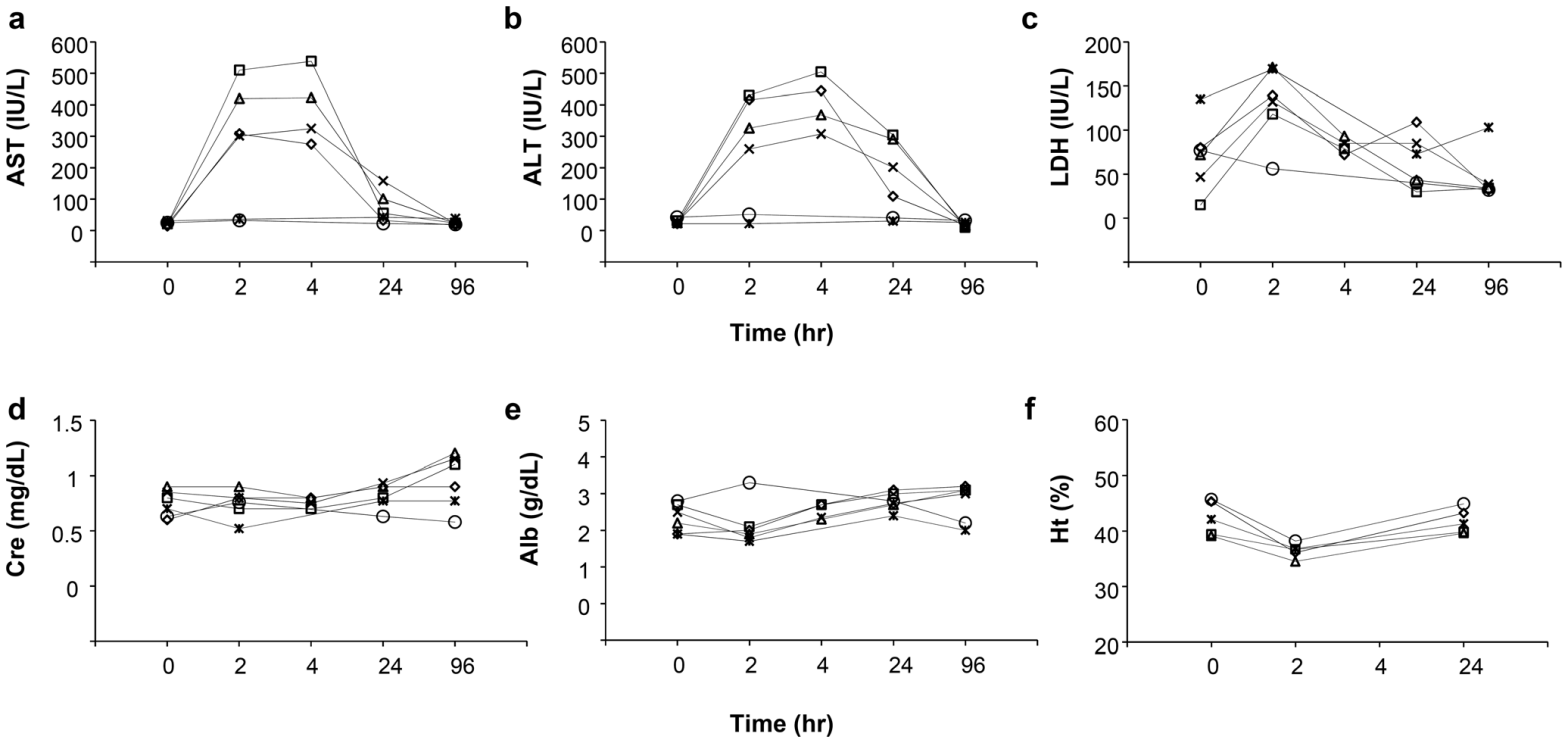
Impact of the procedure on serum biochemistry. Blood samples were collected from the cephalic or saphenous veins of dogs before (time  = 0), and 2, 4, 24, 96 h after the first hydrodynamic injection of saline (white diamond), pCAG-hAAT (white square), pBS-HCRHP-FIXIA (white triangle), pCMV-Luc (black cross) or slow drip infusion of the same volume of saline from peripheral vein within 1 h (white circle) or 2 h (black asterisk). Concentrations of aspartate aminotransferase (AST) (**a**), alanine aminotransferase (ALT) (**b**), lactate dehydrogenase (LDH) (**c**), creatinine (Cre) (**d**), albumin (Alb) (**e**), and hematocrit value (**f**). The values represent mean for hydrodynamic injection groups (n = 2 for each group).

To examine the potential systemic and/or liver-specific toxicity of liver-targeted hydrodynamic gene delivery, various serum biomarkers were analyzed before and after hydrodynamic injection at appropriate time points and compared to those with slow injection from the peripheral vein. Different plasmids were used including pCAG-hAAT, pBS-hFIX, or pCMV-Luc to see whether DNA or DNA sequence makes a difference. Serum analysis showed a transient, 10- to 20-fold increase in hepatobiliary enzymes, including aspartate aminotransferase (AST) (**Figure 5a**), alanine aminotransferase (ALT) (**Figure 5b**), and lactate dehydrogenase (LDH) (**Figure 5c**) after hydrodynamic injection of either saline or saline containing plasmid DNA. In contrast, no changes were seen systemically injected dogs. Enzyme levels returned to normal ranges within 96 h after injection, and serum components, such as creatinine (**Figure 5d**), albumin (**Figure 5e**), blood urea nitrogen, sodium, chloride, and potassium, showed no change (data not shown). Hematogram showed mild decrease of hematocrit after the injections that returned to the background level in 24 h (**Figure 5f**).

**Figure 6:**
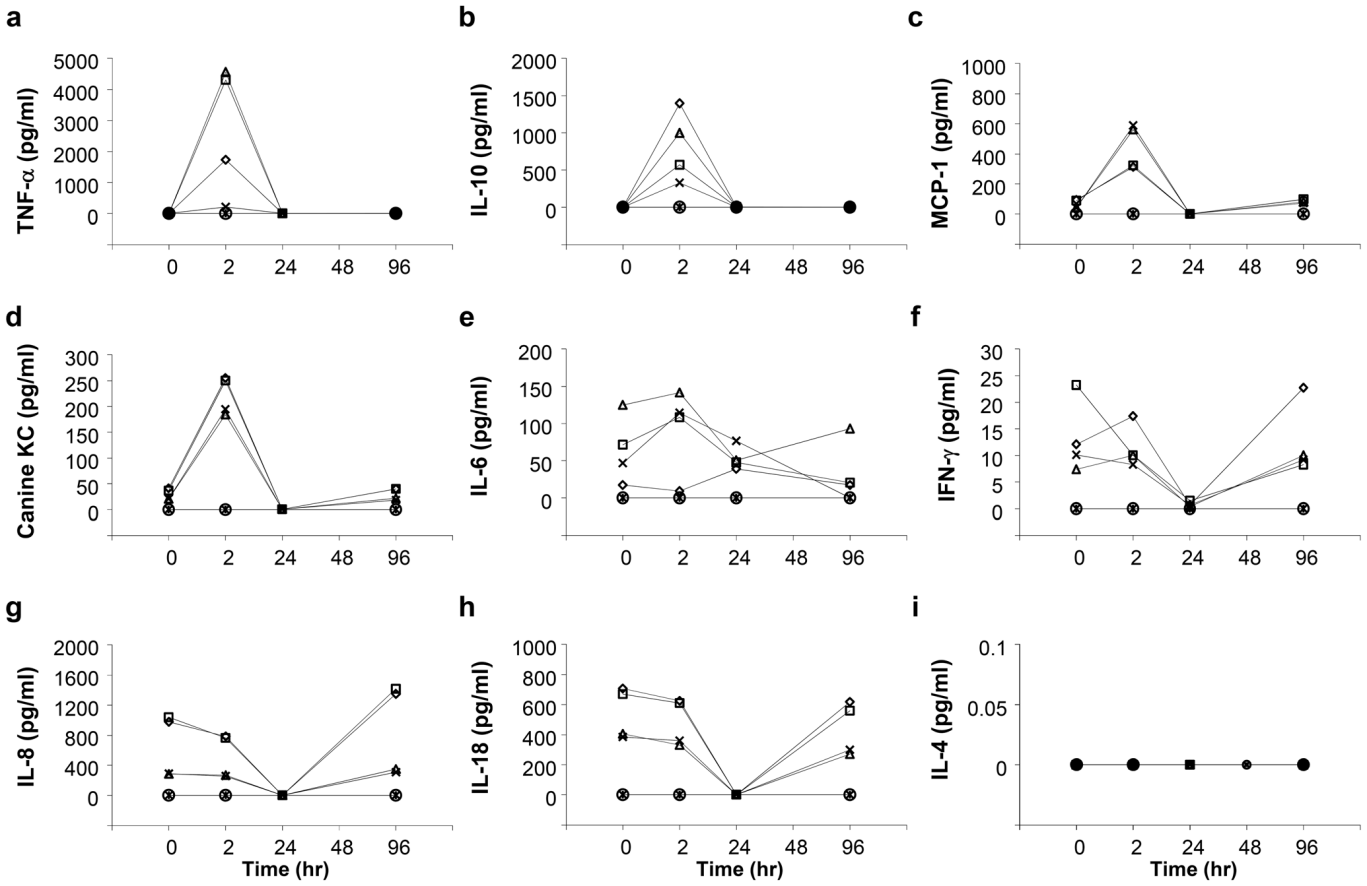
Impact of the procedure on serum cytokine levels. Blood samples were collected from the cephalic or saphenous veins of dogs before (time  = 0), and 2, 24, 48, 96 h after the first hydrodynamic injection of saline (white diamond), pCAG-hAAT (white square), pBS-HCRHP-FIXIA (white triangle), pCMV-Luc (black cross) or slow drip infusion of the same volume of saline from peripheral vein within 1 h (white circle) or 2 h (black asterisk). Concentrations of TNF-α (**a**), IL-10 (**b**), MCP-1 (**c**), Canine KC (**d**), IL-6 (**e**), IFN-γ (**f**), IL-8 (**g**), IL-18 (**h**), IL-4 (**i**). The values represent mean (n = 2 for each group).

Results in **Figure 6** show a significant and transient increase in serum concentrations of tumor necrosis factor-α (TNF-α) (**Figure 6a**), IL-10 (**Figure 6b**), monocyte chemotactic protein-1 (MCP-1) (**Figure 6c**), and Canine KC (chemokine CXCL1) (**Figure 6d**) and a mild increase in IL-6 (**Figure 6e**). A decrease in interferon-γ (IFN-γ) (**Figure 6f**), IL-8 (**Figure 6g**), and IL-18 (**Figure 6h**)**,** and no change in IL-4 level (**Figure 6i**) were seen. The concentrations returned to the background level within 96 h. As IFN-γ, IL-8, IL-18, and IL-4 are known as systemic inflammatory cytokines; TNF-α, IL-10, and MCP-1 relating to myokines for blood vessel; and canine KC relating to wound healing, these results suggest that liver-targeted hydrodynamic gene delivery has no effect on the systemic inflammatory response, and its impact is localized in the injection area, mainly due to vascular stretching.

## Discussion

Hydrodynamic gene delivery has been extensively studied in mice. While effective and safe in small animals, the major challenge is in applying this simple procedure in human gene therapy. For this purpose, a few groups have reported some success in rabbits and small pigs (<20 kg) [8], [9], [21]–[26]. We have reported catheter-based, regional hydrodynamic gene delivery to transfer reporter plasmids to a specific region of a target organ by inserting the catheter into a selected blood vessel [8], [9], [11]. A similar procedure has been used for injecting helper-dependent adenoviral vectors into the livers of non-human primates from the hepatic artery with venous occlusion (so called pseudo-hydrodynamic delivery) [27]–[29]. In addition, the catheter-based procedure with high gene delivery efficiency has been reported in large pigs (40–60 kg) for the liver [8], [11] and muscle [9]. These results suggest that catheter-based regional hydrodynamic gene delivery has a great potential in treating diseases that cannot be treated with conventional therapy. Since early studies focused on the effectiveness of the procedure [8], [9], [11], a thorough analysis of the safety of the procedure is needed. Therefore, in this study, we aim to perform a more thorough assessment of the safety from a clinical point of view. We demonstrated that only transient effects involving vascular stretching and enlargement of the sinusoidal structures upon hydrodynamic injection are detected.

No negative impacts on animals were seen with respect to systemic circulation, respiratory, and cardiac function in dogs who underwent liver-directed gene delivery to multiple liver lobes for 3 times with 2-week interval in between (**Figures 1** **,** **2**). In addition, no damage to the vascular system was evidenced by fluoroscopy before and after the 2^nd^ and 3^rd^ injection suggested the safety of repeated procedure on the vascular system. However, a slight decrease in body temperature was noted during the injection, suggesting that the saline, the carrier solution for plasmid DNA, should be kept warm throughout the procedure. No procedure-related changes in body weight (**Figure 1c**) were observed. These results suggest that the impact of liver-targeted, regional hydrodynamic gene delivery is safe, and the transient effects subside within a few days.

Enlargement of the sinusoids shown in H&E staining (**Figure 3**), the transient decrease in ISO_2_, and the maintenance of IHB (**Figure 4**) provide additional evidence in support that the impact of the liver-targeted hydrodynamic procedure is localized in the liver as evidenced in expansion of the sinusoids in the injected liver. This impact led to a transient increase in serum concentration of hepatic enzymes, while no changes were seen in the other-organ related enzymes such as creatinine, albumin (**Figure 5**), and creatine phosphokinase (data not shown). These results suggest that liver specific hydrodynamic gene delivery transiently enlarged the sinusoids for successful gene delivery and recovered within a short period of time.

A novel analysis was performed in these dogs to monitor serum levels of cytokines. Importantly, no increase in systemic inflammatory cytokines was observed. However, an increase in cytokines related to the myocytes and vascular stretching including TNF-α, IL-10, MCP-1, and Canine KC, was observed. The increase in TNF-α has been reported as a sign of vascular stretching by acute volume loading in dogs [30]. IL-10, a known anti-inflammatory cytokine [31]; MCP-1, a known angiogenic cytokine [32], [33]; and canine KC, a known chemokine (C-X-C motif) ligand 1 (CXCL1); have been reported to respond to balloon injury in rats vessels [34]. On the other hand, no effect was seen in the level of the cytokines related to the systemic inflammatory response including IFN-γ, IL-6, IL-8, IL-18, and IL-4 (**Figure 6**). These cytokines are related to the innate immune responses that have been reported to increase after tail vein hydrodynamic injection in mice [35], [36]. These results suggest that the impact of the liver-targeted hydrodynamic gene delivery in large animals indices enlargement of sinusoids by stretching of the endothelium in the liver.

In summary, the evidence of the safety of liver-targeted hydrodynamic gene delivery has been collected and is presented. Unlike systemic gene delivery through the tail vein in mice, region-specific hydrodynamic gene delivery generates only a transient and site-specific impact and therefore, can be a therapeutic option in human gene therapy from the clinical safety point of view. Further studies to optimize the procedure and injection device, the preparation of plasmid DNA using cGMP procedure, and the minute fine-tuning of the hydrodynamic parameters to satisfy the needs of clinical application will likely bring the use of hydrodynamic gene delivery closer in treating human diseases.
